# Assessing the clinical benefit, safety, and patient-reported outcomes with the use of the PAHcare™ digital platform in pulmonary arterial hypertension: a pilot study

**DOI:** 10.3389/fpubh.2024.1335072

**Published:** 2024-02-16

**Authors:** Gregorio Pérez Peñate, Nuria Ochoa Parra, Juan Antonio Domingo Morera, Amaya Martínez Meñaca, Marta López Ramón, Sergio Cadenas Menéndez, Fernando León Marrero, Sara Gómara de la Cal, Cristina Ghadban-Garrido, Patricia Royo Tolosana, Javier Martín Puentes, Rebeca Aldonza Aguayo, Hadis Mahdavi, Gabriela Bacchini Jeanneret, Pilar Escribano Subías

**Affiliations:** ^1^Unidad Multidisciplinar Vascular Pulmonar, Servicio de Neumología, Hospital Universitario de Gran Canaria Doctor Negrín, Las Palmas de Gran Canaria, Spain; ^2^Centro de Investigación Biomédica en Red Enfermedades Respiratorias (CIBERES), Instituto de Salud Carlos III, Madrid, Spain; ^3^Unidad Multidisciplinar de Hipertensión Pulmonar, Servicio de Cardiología, Hospital Universitario 12 de Octubre, Madrid, Spain; ^4^Instituto de Investigación Sanitaria Hospital 12 de Octubre (i+12), Madrid, Spain; ^5^Servicio de Neumología, Hospital Universitario Miguel Servet, Zaragoza, Spain; ^6^Servicio de Neumología, Hospital Universitario Marqués de Valdecilla, Santander, Cantabria, Spain; ^7^Servicios de Neumología y Cardiología, Unidad de Hipertensión Pulmonar, Complejo Asistencial Universitario de Salamanca, Salamanca, Spain; ^8^Clinical Research Department, Ferrer, Barcelona, Spain; ^9^Digital Health & Technology, Ferrer, Barcelona, Spain; ^10^Corporate Medical Department, Ferrer, Barcelona, Spain; ^11^Centro de Investigación Biomédica en Red Enfermedades Cardiovasculares (CIBERCV), Instituto de Salud Carlos III, Madrid, Spain

**Keywords:** pulmonary arterial hypertension, digital intervention platform, mobile health (mHealth), electronic patient-reported outcome, health services research, patient support program

## Abstract

**Introduction:**

Digital health interventions, particularly mobile health platforms, have shown promise in supporting patients with respiratory conditions, but their application in pulmonary arterial hypertension (PAH) remains limited. We aimed to assess the feasibility, acceptability, and potential clinical benefit of the novel PAHcare™ digital platform as a patient-centred intervention for PAH management through a prospective, single-arm, multicenter pilot study conducted on 53 patients diagnosed with PAH who used the platform for 6 months.

**Methods:**

The primary objective was to assess the impact on Health-Related Quality of Life (HRQoL) through questionnaires. Secondary objectives included evaluating clinical outcomes, including disease progression, PAH signs and symptoms, the 6-min walking test, and the patient’s symptom perception. Additionally, we assessed patient satisfaction and engagement with the PAHcare™ platform, interaction with health coaches, retention, costs and healthcare resource utilisation (HCRU), and safety through monitoring device incidents.

**Results:**

Minimal changes in HRQoL and clinical outcomes were observed over 6 months. A noteworthy 92.4% of patients actively used the platform in the first month, maintaining high usage throughout the study. Patient satisfaction was substantial, with more than half of the patients expressing excellence in service quality, willingness to reuse the platform, and fulfilment of their needs. Health coach interaction was high, with 76% of patients initiating contact within the first week. User retention rates were 70%, with prevalent ongoing usage and interaction with healthcare professionals even after the study. In terms of HCRU and costs, the study showed no significant changes in PAH-related hospital admissions, clinical visits, or tests. Finally, the low number of device-related incidents indicated platform safety.

**Conclusion:**

This pilot study provides compelling evidence supporting the feasibility and acceptability of the PAHcare™ digital platform to empower patients to manage their disease and significantly enhance their overall experience with PAH.

## 1 Introduction

Pulmonary arterial hypertension (PAH) is a debilitating and progressive, rare microvascular disease characterised by elevated pulmonary artery pressure, eventually leading to right heart failure (HF) and death, with reported 1 year survival ranging from 67 to 99% worldwide (1, 2). Besides, this condition imposes a substantial burden on individuals and healthcare systems. On the one hand, PAH significantly reduces the patients’ health-related quality of life (HRQoL), limiting their physical activity and affecting daily functioning (3). On the other hand, the disease often requires complex and costly medical management, frequent hospitalisations, and ongoing monitoring (4). Moreover, the burden extends beyond physical health and seriously impacts emotional well-being, requiring significant adjustments in daily life (3).

Effective management of PAH requires a comprehensive approach where close interaction between patients and healthcare professionals plays a pivotal role in ensuring regular monitoring, adherence to treatment regimens, and implementing necessary lifestyle modifications (5). Moreover, the active engagement of patients in shaping decisions regarding their health conditions is progressively gaining significance (6, 7). An illustrative example is the active participation of patients in the development of the European Society of Cardiology (ESC) and the European Respiratory Society (ERS) clinical guidelines (5, 8). In order to attain this objective, two indispensable elements are health education and the patient’s unique perspective.

Digital health interventions, including mobile health (mHealth) platforms, have emerged as promising tools to support patients and their care teams in the routine management of chronic respiratory diseases such as asthma and chronic obstructive pulmonary disease (COPD) (9–12). Furthermore, a plethora of digital health applications have emerged to enhance HF care throughout the entire spectrum of the HF disease process, encompassing primary prevention, early detection, disease management, and the reduction of related morbidity (13, 14). Digital tools and platforms have the potential to significantly impact patient outcomes by providing continuous support, personalised education, and real-time monitoring (15, 16). Additionally, they can enhance treatment adherence and empower patients by offering access to resources, fostering self-management, and facilitating regular communication with healthcare providers (15–17). Despite their proven efficacy in various healthcare contexts, limited attention has been given to the utilisation of such interventions in the context of PAH. Moreover, existing studies in PAH have predominantly employed electronic health devices, such as wireless or portable tools, mainly focusing on clinical outcomes or their accuracy and reliability, while often neglecting the assessment of health-related QoL (HRQoL) (18, 19).

The PAHcare™ digital platform is a novel mHealth intervention designed to adopt a patient-centred approach to PAH care (20). This innovative platform offers a variety of features, including symptom tracking, educational materials, medication reminders, and effective communication channels with healthcare professionals (20). By granting convenient access to educational resources, facilitating seamless communication with healthcare professionals, and promoting self-care behaviours, the platform possesses the potential to empower patients and augment their active involvement in their own care.

In this manuscript, we present the findings of the pilot study conducted to evaluate the clinical benefit and safe use of the PAHcare™ digital platform in patients with PAH. The study quantitatively assessed health-related quality of life (HRQoL), disease severity, disease-related signs and symptoms, cost and healthcare resource utilisation, as well as patient engagement and satisfaction with the PAHcare™ platform.

## 2 Methods and analyses

### 2.1 Study design

This prospective, single-arm, multicenter pilot study was designed to evaluate the safety, feasibility and potential clinical benefits of the innovative PAHcare™ digital platform in patients with PAH and their care team over 6 months (20). The study was conducted at five specialised PAH units of reference hospitals within the Spanish public healthcare system (Supplementary Table S1).

### 2.2 Ethical considerations

The study was conducted in accordance with the Declaration of Helsinki, the international standard ISO 14155 “Good Clinical Practice” guidelines, and the European and Spanish Regulations on Medical Devices (21, 22). All patients gave written informed consent to participate in the study. The competing Ethics Committee of Hospital 12 de Octubre, Madrid, Spain, approved the study protocol V4.0 on March 22, 2022 (FPAH-CI-2101).

### 2.3 PAHcare™ digital health platform overview

The CBS-PAH study’s protocol, previously published, provides details of the PAHcare™ digital platform (20), which consists of a patient-oriented mobile app and dedicated dashboards for physicians and health coaches (HCs). Briefly, it enables users to log patient-reported outcomes (PROs) data, including symptoms, medication-related adverse events, hospitalisations, clinical information, and lifestyle details. Besides, the app provides evidence-backed content through magazine articles, frequently asked questions (FAQs), lessons, and quizzes. Additionally, users receive medication reminders and personalised support through chats and calls with specifically trained HCs offering assistance tailored to individual treatments. For those on the prostacyclin analogue treprostinil, HCs can assist with treatment, medical device support, adverse event reporting, and provide advice on platform use, self-monitoring, lifestyle changes, behavioural modifications, and symptom management. Patients on other treatments receive HC support focused on platform usage and overall health management. Lastly, an external portal offers additional psychological support for treprostinil patients as needed.

### 2.4 Population

All enrolled patients were adults aged 18 years or older with a confirmed diagnosis of PAH, regardless of disease severity (World Health Organization [WHO] functional class I to IV), who were considered suitable for participation in the self-care/caregiver-driven support program for PAH based on the investigator’s judgement. Additionally, participants were required to provide signed informed consent and be able to read, speak, or understand Spanish.

Exclusion criteria included any psychological and/or physical conditions that could negatively affect the proper adherence to study procedures (e.g., uncorrected hearing and/or visual impairments) or the absence of a smartphone for accessing the program. Additionally, patients who had undergone a major surgical intervention within the 30 days prior to enrollment or those experiencing complications that could hinder the complete utilisation of the patient support program were excluded. Lastly, pregnant women, lactating or planning to become pregnant within the subsequent 6 months, were not eligible for study participation (20).

### 2.5 Study procedures and outcome measures

Enrolled patients underwent a baseline visit and two follow-up visits 3 and 6 months after the initial visit. As part of the onboarding process, patients were instructed to download the mobile app [a complete description of the app’s features can be found in the previous publication of the CBS-PAH study protocol (20)]. With the assistance of their assigned health coach (HC), patients configured the app and provided their basic personal information, medical history, and lifestyle details.

The primary objective of the CBS-PAH study was to assess the impact of PAHcare™ intervention on patient’s HRQoL. To evaluate this, participants completed two different questionnaires at each visit. The first questionnaire used was the Spanish-validated version of the emPHasis-10, which explicitly measures HRQoL in PAH (23, 24). It focuses on breathlessness, fatigue and lack of energy, social restrictions, and concerns regarding effects on patients’ significant others, including family and friends. Each item is scored on a semantic differential six-point scale (0–5) using contrasting adjectives at each end. The total emPHasis-10 score ranges from 0 to 50, with higher scores indicating a worse quality of life. The second questionnaire employed was the Spanish-validated version of the Euroqol 5-dimension questionnaire (EQ-5D-5L) (25, 26). The EQ-5D-5L consists of five dimensions (mobility, self-care, usual activities, pain/discomfort, and anxiety/depression), each with five levels of severity (ranging from no problems, slight problems, moderate problems, severe problems and unable to/extreme problems) scored from 1 to 5, respectively. Lastly, the EQ-5D-5L also includes a visual analogue scale (VAS), where responders mark with an X on a 20 cm scale with endpoints labelled “Best imaginable health state” (100) and “Worst imaginable health state” (0) their health that day.

As a secondary objective, we determined the impact of the PAHcare™ intervention on the clinical progression of the disease. This was assessed by analysing clinical parameters recorded at each visit. These parameters included the severity of the disease on the WHO functional classification, as well as the presence, frequency (times/day and/or week), and intensity (mild, moderate, or severe) of PAH signs and symptoms (such as dyspnoea, orthopnoea, fatigue, syncope, chest pain, and oedema), 6-min walking test (6MWT) and patient’s PAH symptoms perception graded on a visual analogue scale (VAS). Furthermore, the study had secondary objectives that focused on evaluating the usage and engagement of the PAHcare™ platform. This was done by analysing the interactions between patients and the platform, including the number and duration of logs for PROs, the consumption of educational content such as magazines, lessons, and quizzes, and the number and duration of calls and completed chats with the health coach (HC). Moreover, the patient’s satisfaction with the use of the PAHcare™ platform was assessed using the 8-item Client Satisfaction Questionnaire (CSQ-8), with each item rated on a 1 to 4 scale (25), completed by the patients after 6 months of using the platform. In addition, the description of the reason, duration and procedures for PAH-associated hospitalisation/visit/emergency room admissions and formal and informal costs related to the disease through an *ad-hoc* questionnaire were also assessed. Lastly, safety was evaluated by monitoring reported device incidents, which referred to any inadequacy of the medical device concerning its identity, quality, durability, reliability, usability, safety, or performance.

### 2.6 Sample size and statistical analyses

Since this pilot study was exploratory, we did not conduct a power analysis to determine a specific difference in the outcomes. Instead, our primary objective was to ensure a minimum sample size of 50 individuals actively utilising the PAHcare™ platform.

The primary and secondary objectives were evaluated in two populations: the full analysis set (FAS), which included all selected patients, and the evaluable population (EP), which included selected patients with available data (baseline and at least one post-baseline assessment in one of the two HRQoL questionnaires) for analysing the primary endpoints. Additionally, the evaluable population per protocol (EP-PP) consisted of patients in the EP who had assessments at baseline and at least one post-baseline evaluation in both HRQoL questionnaires within the specified window of ±15 days as defined in the protocol. The cost evaluable population (CEP) included all patients with data available in baseline and 6 months who answered at least 10 questions of the 20 performed related to health costs and resources.

All variables were analysed descriptively at each visit, with categorical variables summarised using absolute and relative frequencies and continuous variables through mean, standard deviation (SD), median, and minimum and maximum values. To compare continuous variables between baseline and different clinical investigation visits, *t*-tests or Wilcoxon test were used, while Mc Nemar’s test was employed for categorical variables. For the primary endpoint (change in the EmPHasis-10 and EQ-5D-5L scores), missing data from post-baseline visits were imputed using the last observation carried forward (LOCF) method. In contrast, no imputation of missing data was performed for secondary variables. The significance threshold was set at a two-sided ⍺ = 0.05. All data processing, summarisation and analyses were performed using the Statistical Analysis System (SAS) statistical software package, version 9.4. SAS Institute Inc., Cary, NC, United States.

## 3 Results

**Figure 1:**
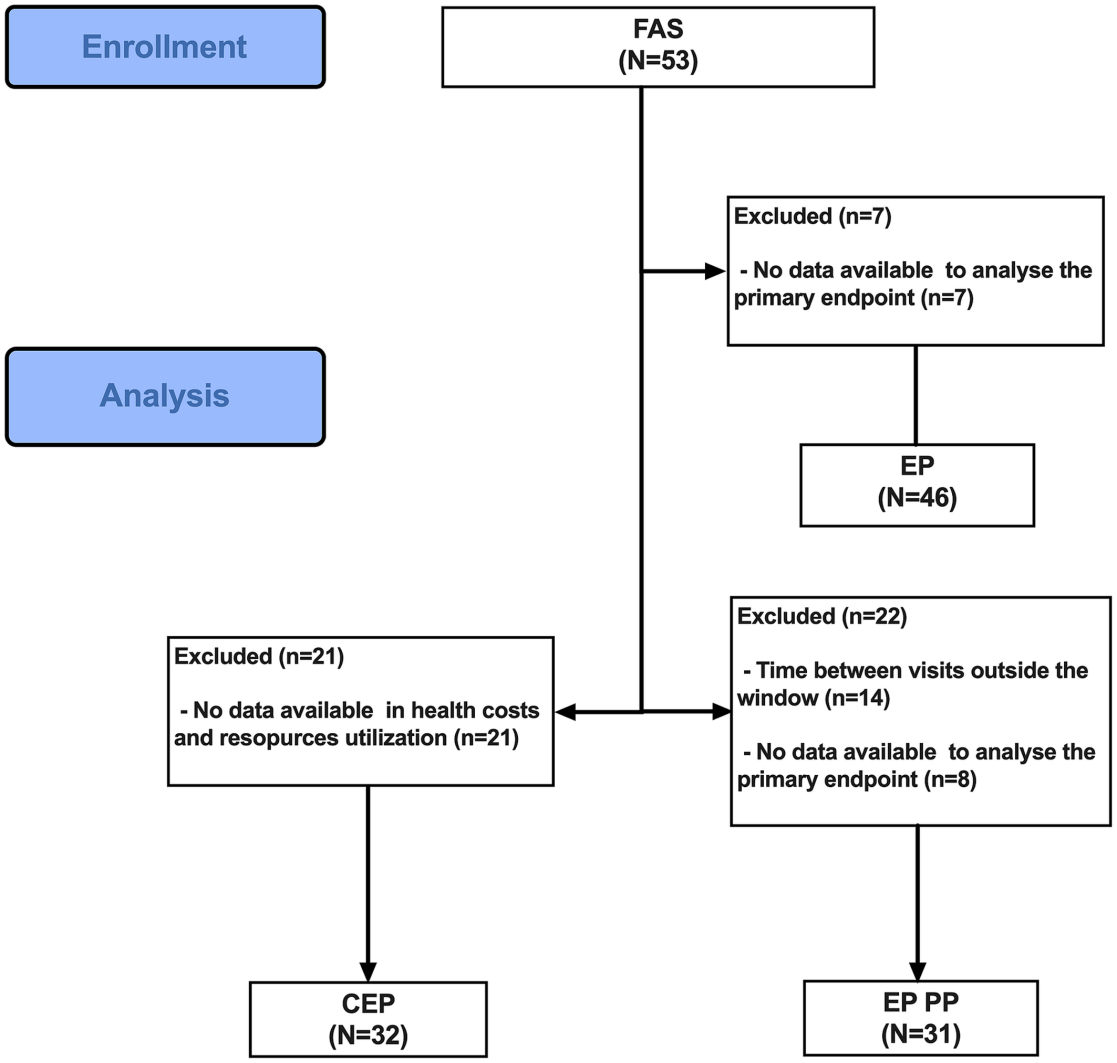
Flow diagram of the CBS-PAH study. CEP, cost evaluable population; EP, evaluable population; EP PP, evaluable population per protocol; FAS, full analysis set.

The study enrolled a total of 53 patients who had a confirmed diagnosis of pulmonary PAH and actively utilised the PAHcare™ platform (Figure 1). Among them, 46 patients constituted the evaluable population (EP) with available data for analysing the primary endpoint. Seven patients discontinued the study prematurely, two withdrew consent, three were lost to follow-up, and two withdrew for other reasons.

At baseline, the mean age of the patients was 48.8 years (SD = 12.6). The majority of participants were female (82.6%), with more than half being classified as WHO functional class II (58.7%), while none were classified as class IV (Table 1). The most commonly reported symptom was dyspnea, reported by 43.5% of patients, with approximately 50% of them experiencing mild intensity.

**Table 1:** Participant’s characteristics.

Characteristic	Patients (*N* = 46)
Age (years) (mean, SD)	48.8 (12.6)
Gender (*n*, %)	
Female	38 (82.6)
Male	8 (17.4)
Body weight (kg) (mean, SD)	66.1 (15.1)
WHO Functional class (*n*, %)	
Class I	17 (37.0)
Class II	27 (58.7)
Class III	2 (4.3)
Class IV	0 (0.0)
6MWD (meters), mean (SD)	486.2 (85.2)
Signs and symptoms, *n* (%)	
Dyspnoea, *n* (%)	20 (43.5)
*Frequency, mean (SD)/day*	2.6 (1.8)
*Mild*	10 (50%)
*Moderate*	9 (45.0)
*Severe*	1 (5.0)
Orthopnoea, *n* (%)	3 (6.5)
*Mild*	2 (66.7)
*Moderate*	1 (33.3)
Fatigue, *n* (%)	12 (26.1)
*Mild*	6 (50)
*Moderate*	6 (50)
Chest pain, *n* (%)	8 (17.4)
*Frequency, mean (SD)/day*	1.5 (0.7)
*Frequency, mean (SD)/week*	1.6 (0.9)
*Mild*	6 (75.0)
*Moderate*	2 (25.0)
Oedema, *n* (%)	5 (10.9)
*Intensity*	
*Mild*	3 (60.0)
*Moderate*	2 (40.0)
Syncopes	0 (0.0)
Hemodynamics as per RHC (*n* = 45), median (Q1, Q3)	
PAP (mmHg)	49.0 (37.0, 60.0)
PCWP (mmHg)	9.0 (7.0, 12.0)
PVR (Wood units)	9.6 (6.3, 15.6)
Right Atrial Pressure (mmHg)	7.0 (5.0, 9.0)
Mixed Venous Oxygen Saturation (%)	67.4 (64.0, 74.9)
Cardiac Index (mL/min/m^2^)	2.5 (2.1, 3.1)

6MWT, 6-min walking test; PAP, pulmonary arterial pressure; PCWP, pulmonary capillary wedge pressure; PVP, pulmonary vascular resistance; RAP, right atrial pressure; RHC, right heart catheterization; WHO, World Health Organization functional class.

### 3.1 Changes in EmPHasis-10 and EQ-5D-5L scores

**Figure 2:**
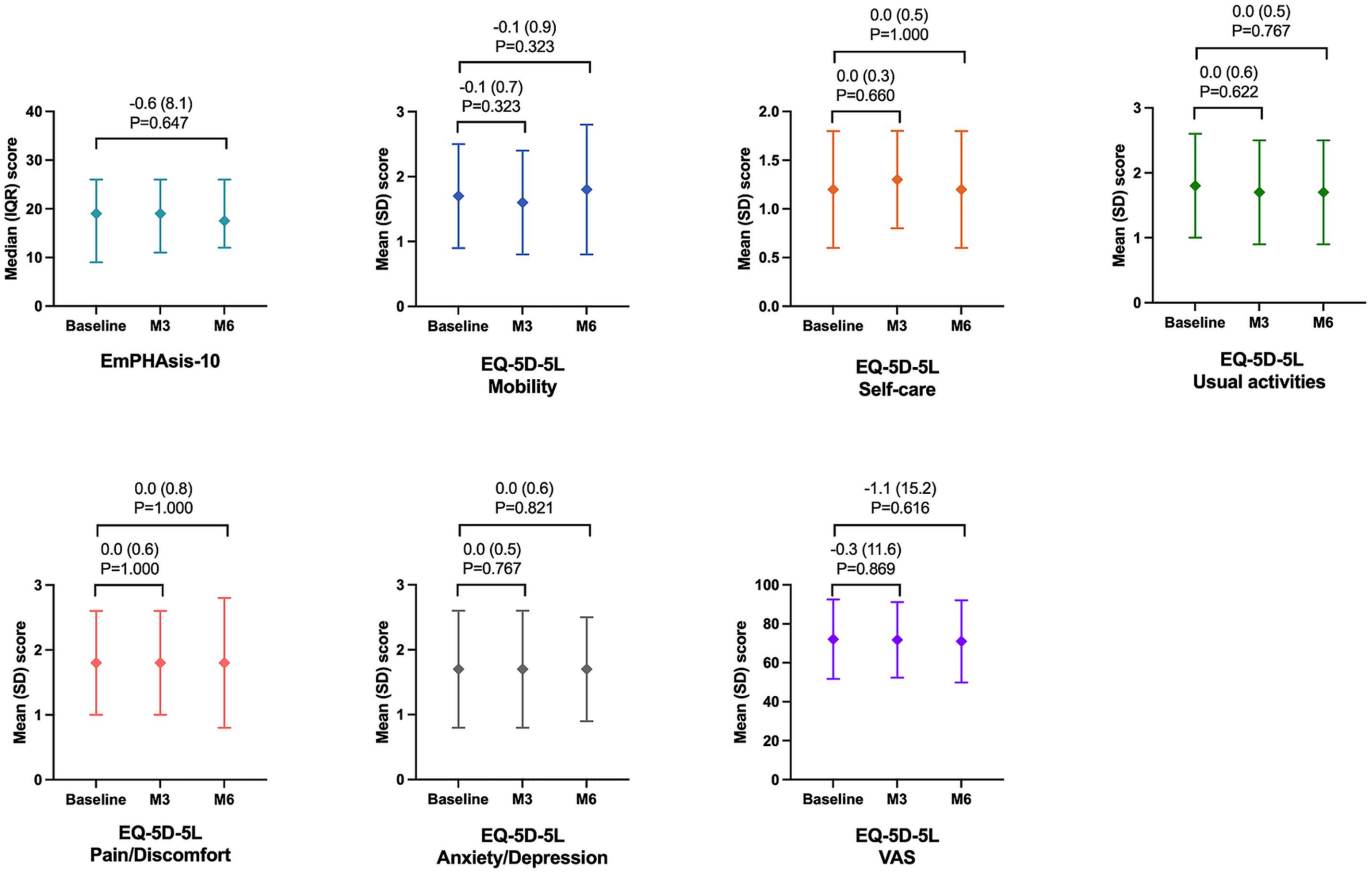
Change in the EmPHAsis-10 and EQ-5D-5L scores during the study follow-up.

At baseline, the mean EmPHasis-10 QoL questionnaire score was 19 (ranging from 9 to 26), which corresponds to an intermediate risk level associated with a 10% likelihood of 1 year mortality (27). Throughout the follow-up period (6 months after the baseline visit), there was a minimal decrease of less than 1 point (−0.6; SD = 8.1), which did not reach statistical significance (Figure 2). As for the EQ-5D-5L, the initial mean scores for all dimensions were between 1 and 2, thus ranging between “no problems” and slight problems,” and the mean EQ-5D-5L VAS at baseline was 72.1 (SD = 20.4). No statistically significant differences were observed between the baseline and final visit for any of the five dimensions of the EQ-5D-5L or the VAS score (Figure 2). The findings were consistent across both the FAS and the EP-PP populations, which did not deviate from the results obtained in the EP.

### 3.2 Change in severity and signs and symptoms of PAH

Most patients (72.7%) remained in the same WHO functional class throughout the study duration. Among those who experienced a change (27.3%), there were five cases (41.7%) showing improvement and seven patients (58.3%) showing deterioration. When considering the 6MWT, the mean distance covered slightly decreased from baseline to the end of the study, but this difference was not statistically significant (difference = −0.2 meters; SD = 45.7; *p* = 0.889). These results were consistent with those observed in both the FAS and the EP-PP populations.

**Figure 3:**
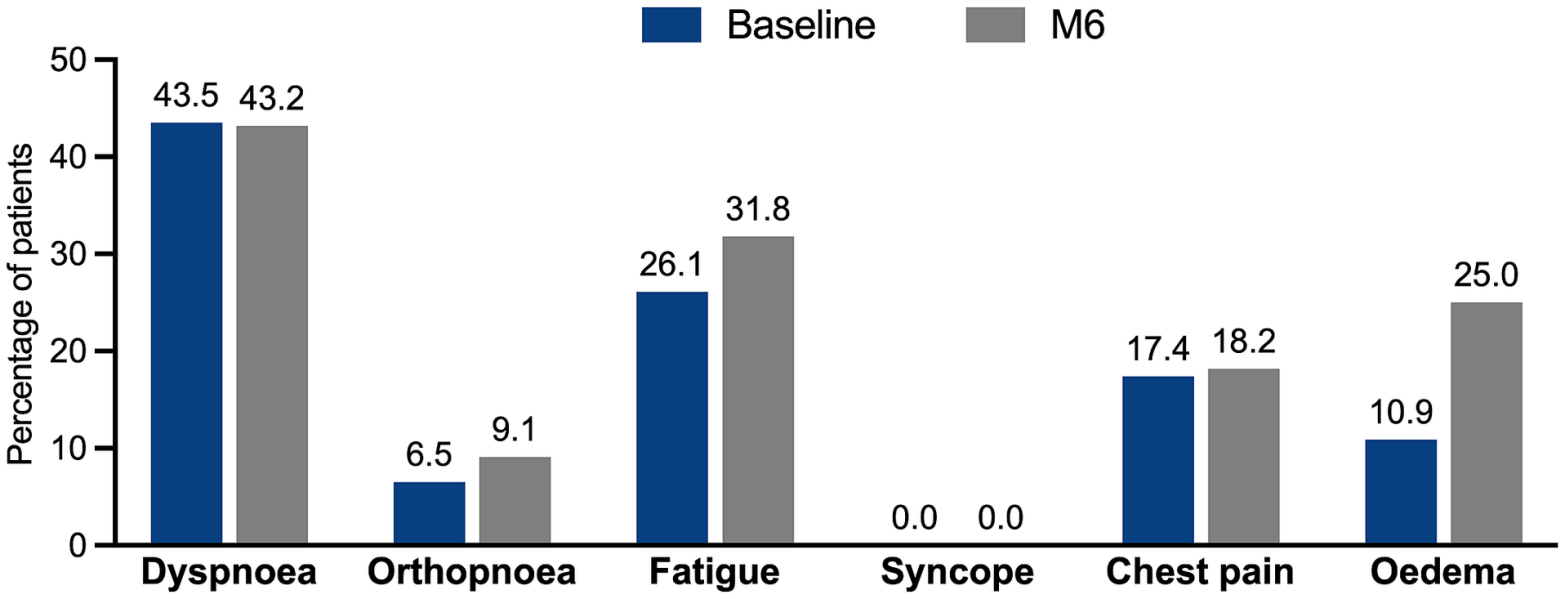
Change in the percentage of signs and symptoms of PAH experienced by the patients throughout the study.

Furthermore, no significant changes were observed in the mean VAS self-rated perception of PAH symptoms, nor in the percentage of patients experiencing different signs and symptoms of PAH (Figure 3). These figures were comparable to those reported in the FAS and EP-PP populations.

### 3.3 Patient-reported satisfaction with the use of the PAHcare™ platform

**Figure 4:**
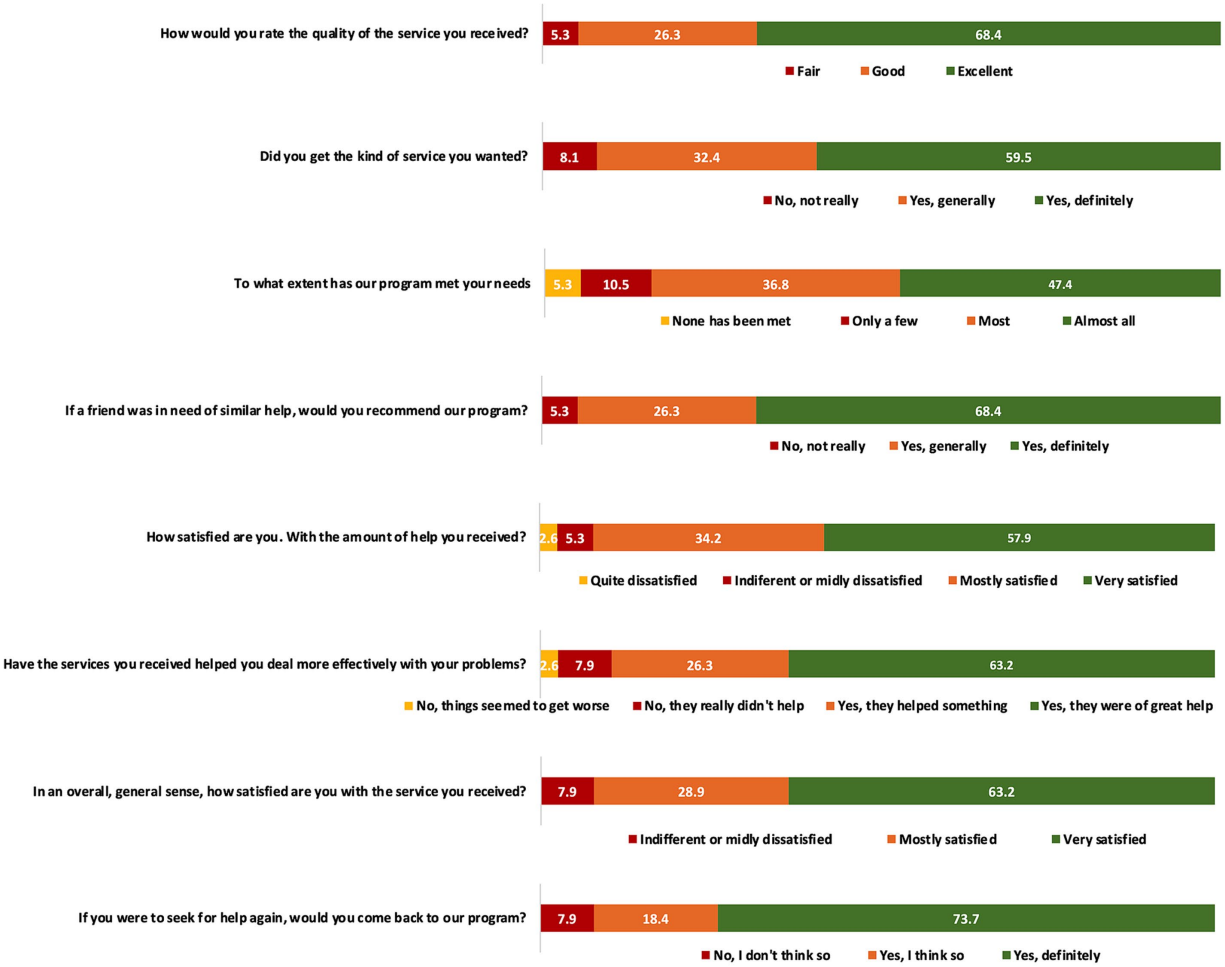
Results of the CSQ-8 questionnaire on the patient’s satisfaction with the use of the PAHcare™ platform.

The responses to the CSQ-8 questionnaire revealed a notable level of satisfaction with the platform, as more than half of the patients assigned the highest score to nearly all the questions (Figure 4). Specifically, 68.4% of patients regarded the quality of the service as excellent, 73.7% expressed their willingness to use the platform again, and 84.2% believed that their needs had been met (with 47.4% specifying that all their needs were met, and 36.8% stating that most of their needs were met).

### 3.4 Consumption of educational content

The proportion of patients engaging with disease-specific knowledge and management magazines is presented in Supplementary Figure S1. Among the articles, the highest readership was observed for the one centred around pulmonary rehabilitation, with 57% of patients accessing it. This was followed by the articles titled “All about PAH” and “Living with a pump,” both accessed by 40% of patients. Additionally, the articles “Coping with cognitive impairment” and “How to accomplish more in 1 day” were accessed by 38% of patients each. The remaining articles had a readership of less than 36% among patients.

Regarding the utilisation of the platform’s structured educational pathway, 45.3% of patients successfully completed Level 1 (Supplementary Figure S2). Afterwards, the proportion of patients progressing to subsequent levels decreased gradually, with 36% of patients completing at least two levels of education and 26.4% successfully completing all four levels.

### 3.5 Adherence, engagement, and retention

**Figure 5:**
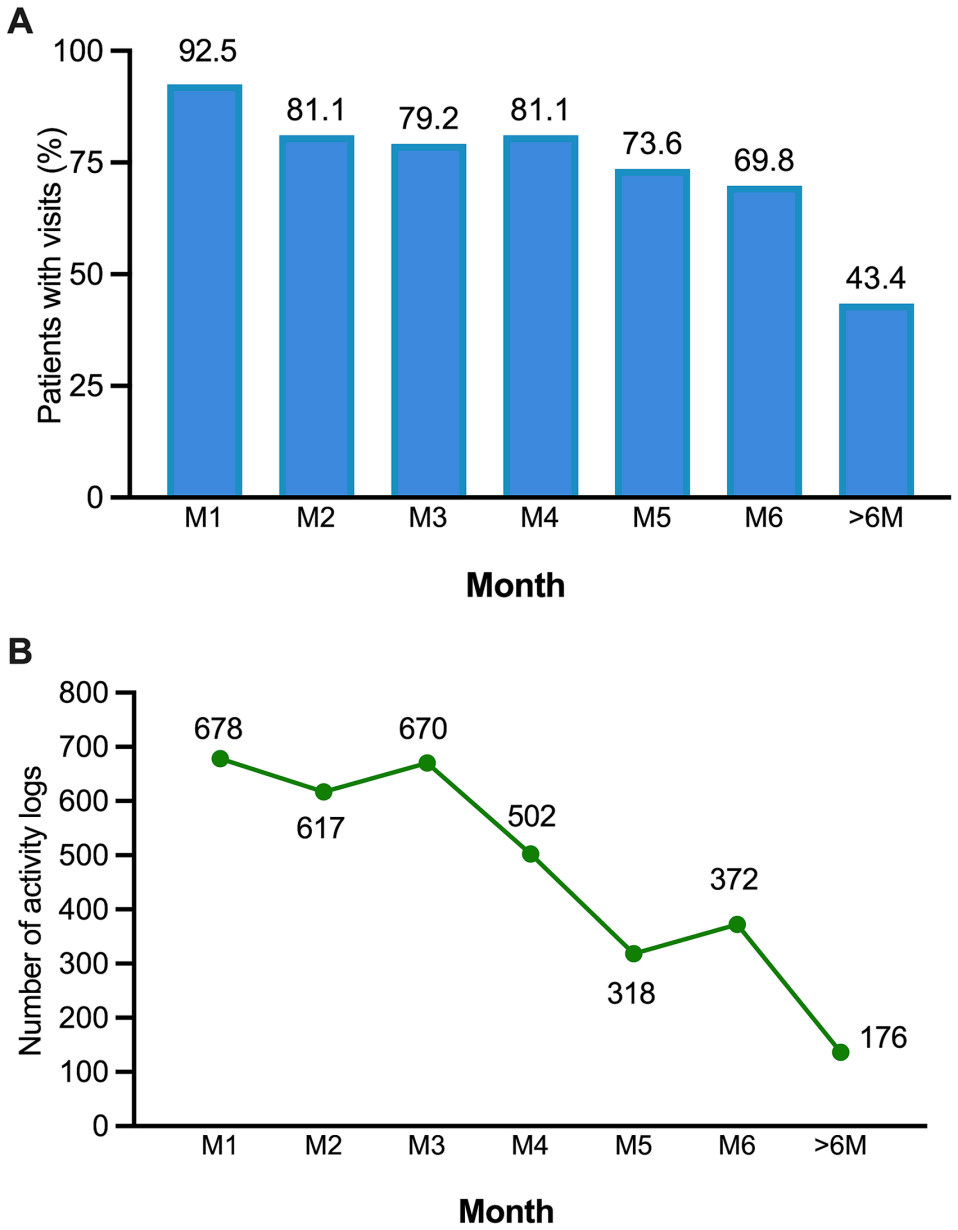
**(A)** Proportion of patients accessing the PAHcare™ platform by month of use. **(B)** Number of activity logs to the platform by month.

During the first month of the study, an overwhelming majority of patients (*n* = 48; 92.4%) actively engaged with the PAHcare™ platform (Figure 5A), resulting in a total of 2,912 recorded visits. On average, each patient launched the platform 215 times, indicating a daily usage rate of at least one launch per patient. Although the proportion of patients accessing the platform gradually decreased over time, it remained notably high at the end of the study (*n* = 37; 69.8% at 6 months). Additionally, nearly half of the patients continued utilising the app even after the study concluded, with 43.4% still accessing it after 6 months of use. Moreover, the logging of activities within the app was extensive, with nearly all patients (*n* = 49; 92.4%) actively using the app’s activity logs (Figure 5B). The most frequently logged activities were meals (mean = 210; SD = 393.6), followed by weight (mean = 46.5; SD = 85.3), water intake (mean = 18.6; SD = 39.1), and blood pressure readings (mean = 18.3; SD: 38.2). A total of 21 patients (39.6%) actively recorded their symptoms assessments within the app, averaging 2.26 days per month (SD = 2.56).

**Figure 6:**
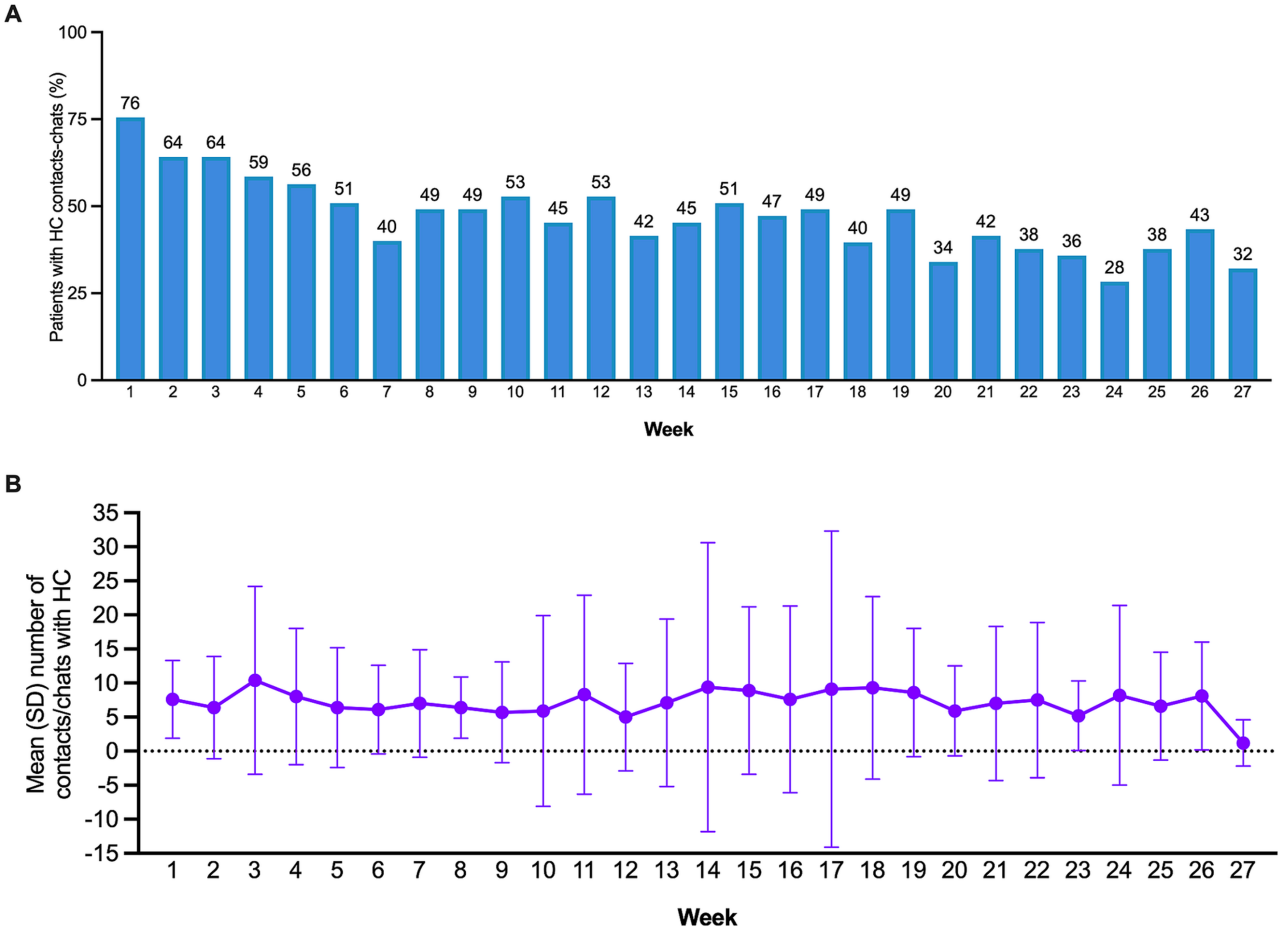
**(A)** Proportion of patients with contacts-chats with the health coach by month. **(B)** Mean number of chat lines per week.

During the initial week of platform usage, 76% of patients opted to contact or chat with their assigned HC (Figure 6A). Although the proportion slightly declined over time, it remained above 50% even after 19 weeks. At the conclusion of the study (24 weeks), 28% of patients still sought HC contact, and following the study’s end, between 38 and 32% continued utilising this feature. On average, patients made 7.6 contacts or chats with HCs (SD = 5.7), maintaining a consistent level throughout the study (Figure 6B). On the other hand, health professionals made an average of 2.9 phone calls to each patient per month (SD = 1.3).

Remarkably, the PAHcare™ platform exhibited outstanding user retention rates, with 79.2% of patients continuing to use it after 3 months. Furthermore, patient retention remained substantial, with rates of 69.8 and 43.4% at 6 months and after 6 months, respectively.

### 3.6 Costs and healthcare resource utilisation

The CEP population consisted of 26 patients at baseline and 32 at the end of the study. No significant changes were observed in the number of PAH-associated hospital admissions (including visits to the emergency room), clinical visits/consultations to health professionals, or the number of tests performed during the 6 months before inclusion and the 6 months lapsed between study initiation and final visits (Supplementary Tables S2, S3).

Noteworthy, 41.9% of patients (*n* = 13) were working at baseline, a number that increased to 46.9% (*n* = 15) at the final visit. On the other hand, only five patients reported a decrease in their regular incomes (16.7% of the baseline sample and 17.9% of the final visit sample).

Three patients (9.4%) at baseline and one patient (3.1%) at the final visit had to buy a medical equipment or device to carry out works to adapt their home to their needs. One patient had to pay himself a wheelchair (2.237 €) and one a dehumidifier (49 €). Three patients at baseline and one at the final visit reported to benefit from help in domestic cleaning (private in all cases; median = 2.0 days). Help from family and friends was available for seven patients at baseline with a median dedication time 25.8 h (IQR:12.9, 51.6) and for six patients at the final visit with a median time of 10.8 h (IQR:8.6, 28.0).

Data available data on formal and informal care costs was not sufficient to conduct a proper economic analysis to assess the impact of the platform on disease-associated costs.

### 3.7 Safety of the PAHcare™ platform

Only 5 (11.1%) patients experienced incidents with the device in the EP population. Those were reported as one case of “frozen app,” two as “it does not work,” and the last one as “no information of all food.” Three of them occurred at the initial use of the medical device. None of them was reported as serious. Safety analysis was comparable in both EP and FAS populations.

## 4 Discussion

We conducted a pilot study to evaluate the clinical benefit and safe use of the PAHcare™ digital platform as a novel, patient-centred mHealth intervention for the routine care of patients with PAH and their care team. To the best of our knowledge, this study is the first one of its kind to be implemented in patients with PAH, making it unique and without any existing benchmarks. After 6 months of utilisation, there were no significant differences compared to baseline in HRQoL measures, disease severity, or disease-related signs and symptoms. However, the study revealed remarkably high levels of engagement, satisfaction, acceptability, and usability of the PAHCare™ platform.

The lack of significant improvements in health-related outcomes observed in our study could be attributed to various factors inherent to the study design. Firstly, due to the low prevalence of PAH, we opted for a single-arm pilot study without a comparison control group. This decision was made in order to maximise the number of patients who could benefit from the PAHcare™ platform intervention. Secondly, in addition to the limited sample size, the study lasted only 6 months, which may have influenced the ability to capture significant clinical changes. Indeed, given the chronic nature of PAH, longer-term interventions may be necessary to observe substantial shifts in parameters such as WHO functional class or 6MWDT. Thirdly, although we intended to include patients from all WHO functional classes, 96% of the enrolled participants were categorised as functional classes I and II. The skewed distribution across WHO functional classes may have had implications for the observed outcomes. The predominance of participants with relatively conserved functional capacity suggests that, at baseline, a majority effectively managed their condition with established coping mechanisms, treatment plans, and lifestyle adjustments. This pre-existing effective management likely contributed to the limited clinical changes observable during the relatively brief study period. Therefore, the possibility of a beneficial clinical effect in patients with lower functional capacity (WHO functional class III and IV) cannot be excluded. In these advanced cases, comprehensive pharmacological and non-pharmacological care has the potential to enhance disease symptoms and improve the overall QoL (5). Undoubtedly, making treatment decisions at these stages poses significant clinical challenges, as patients often suffer from psychological comorbidities such as depression and anxiety, conditions that substantially diminish the patient’s HRQoL (28–30) and can also detrimentally affect treatment adherence and disease progression (31). Although we did not identify significant changes in the anxiety/depression dimension of the EQ-5D-5L questionnaire from baseline to the study’s conclusion, we acknowledge the importance of a thorough evaluation of additional psychological factors. These could include aspects such as social support, self-efficacy, coping mechanisms, and the patient’s confidence or perceived control over their health. Given the remarkable levels of engagement, retention, and satisfaction observed with the PAHcare™ digital platform, it is conceivable that a more comprehensive assessment of psychological factors might have revealed significant differences. In addition to the prevalence of patients experiencing only mild disease symptoms, it is noteworthy that the studied subjects exhibited a relatively high QoL at the outset of the study. However, our study lacks comprehensive data on comorbidities, such as ischaemic heart disease, hypertension, or sleep apnoea, which are known to adversely affect the overall HRQoL in individuals with PAH (3, 32). Lastly, although we do not have data on the pharmacological treatments, it is important to highlight that all patients in our study were treated at specialised PAH units in reference hospitals in Spain and had already stabilised their condition with a well-adjusted care and treatment plan. PAH requires a high level of expertise from healthcare providers, and clinical guidelines advocate for the referral of patients to Speciality Care Centres (SCCs) for optimal management (5, 33, 34). This approach has been consistently associated with improved patient outcomes, including reduced hospitalisations and mortality rates (35). In Spain, it has been estimated that there are currently 30–35 active PAH units (36). A recent survey conducted among physicians involved in the management of PAH revealed considerable variation between SCCs in terms of organisational models, diagnostic resource availability, and adherence to clinical practice guidelines (36). Furthermore, the survey highlighted several structural deficiencies within the PAH units. These included inconsistencies in the percentage of patients receiving health education, inadequate attention given to QoL assessments, and a frequent lack of access to support from social workers or psychologists (36). Additionally, it should not be extended to non-specialist PAH care centres that may possess varying levels of PH expertise and PH-specific resources, diverse multidisciplinary care approaches, different access to advanced diagnostic tools, availability of advanced therapies, distinct referral pathways, or supportive services (such as emotional support, disease management education, and guidance for coping with the challenges associated with living with PAH).

In addition to the essential objectives of promoting symptom monitoring, control, and improving the patient’s QoL, mobile devices offer numerous additional benefits that are challenging to quantify but hold significant value for the healthcare system, providers, and patients. For instance, real-time communication between healthcare professionals and patients enhances patient engagement, promotes shared decision-making, and fosters collaborative care (6). Moreover, access to health education materials and self-management tools empowers patients to actively manage their disease (37, 38). In our study, we observed a notable level of app usage, with approximately half of the patients actively engaged with the chat/contact feature with their assigned HC throughout the follow-up period. Furthermore, 32% of the patients were still actively messaging their coach for support even after the study concluded. Patients also displayed significant interest in furthering their understanding of PAH by accessing magazines and educational programs offered on the platform. Lastly, the PAHCare™ platform received positive feedback from patients, with a high level of program satisfaction reported. Specifically, 92.1% of patients expressed satisfaction (either generally satisfied or definitely satisfied), and 84.2% felt that their needs had been effectively addressed. This underscores not only the patient’s evident desire for comprehensive information and support but also demonstrates the platform’s ability to effectively meet those needs, thereby facilitating a better understanding of the healthcare process and ultimately enhancing the overall patient experience.

All of the aforementioned factors probably played a crucial role in the platform achieving higher retention rates compared to other mHealth applications. Remarkably, studies indicate that approximately 80% of participants in mHealth interventions for chronic diseases only engage at a minimal level and fail to sustain long-term usage (39). Indeed, recent estimates suggest that approximately 72% of subjects launch healthcare applications five times or less, while 17% use them ten times or less within a span of 30 days (40). In contrast, patients used the PAHCare™ app on average at least once a day. Additionally, it has been estimated that the average 30 days retention rate for medical apps is 52% (79.2% in our study), dropping to 31–39% (69.8% in our study) after 90 days (41). A recent systematic review conducted to examine factors influencing adherence to mHealth apps found that personalised features such as tailored feedback and needs, along with ease of use and direct communication with healthcare professionals, positively influenced adherence to respiratory disease management applications (24). Additionally, the review found that adherence scores were significantly higher for apps provided exclusively as part of scientific studies, as opposed to those publicly available through app stores (39). The review also demonstrated that user engagement was higher for apps developed by private app development companies compared to those created by public institutions or research groups (39). All these factors probably contributed to the attractiveness of the PAHCare™ platform and the sustained engagement and high retention rates observed in our study.

A recent review showed that Patient Support Programs (PSPs) designed with the aim of improving adherence and patient empowerment induced a positive impact on patient’s adherence to medication, patient satisfaction, and HRQoL, and additionally showed that home therapy led to substantial cost savings (42). The main objectives of PSP are to ensure the correct delivery and management of medication, to improve healthcare professional’s training and knowledge on treatment management, and to deliver very close patient care. In some countries, all this is taken care of by the PAH healthcare team, mainly by the specialised nurses, but in some cases externally provided additional support is needed. Mobile health platforms such as PAHcare can be used to deliver this patient support to patients in a straightforward and efficient manner. However, the observed increase in activity logs during the months corresponding to clinical follow-up appointments raises an interesting point. It is plausible that the additional motivation stemming from personal contact during these scheduled appointments contributed to heightened engagement with the PAHcare™ platform. Thus, the interaction with healthcare professionals during clinical follow-ups may have served as a catalyst, prompting users to actively participate and log more information on the platform. This finding suggests the potential synergistic effect of combining digital interventions with traditional, in-person clinical interactions.

Healthcare research requires different methodological approaches, such as qualitative and quantitative analyses, to understand the phenomena under study. Central elements of the qualitative method are that the object of study is constituted by perceptions, emotions and beliefs (43). Having seen that the high level of satisfaction shown by the study participants was not translated into a direct clinical benefit, the information gathered through the qualitative methodology may facilitate the understanding of critical points, barriers and facilitators that contribute to better management of PAH, considering the perspective of the patients and the health care team. Indeed, patient engagement goes beyond traditional activities in healthcare and extends to involving patients in designing and implementing care delivery systems, shaping health policies, and directing health research (6). In this context, the PAHcare™ digital platform emerges as a tool to collect patients’ insights and experiences. These insights are crucial for aligning healthcare systems with the patient’s needs, priorities, and preferences, ultimately enabling their active participation in governance decisions and defining management strategies. However, it’s imperative to recognise that personalised support and user engagement in the context of mHealth apps also extends to privacy concerns and the willingness to share personal information (44). Thus, the development of a trustworthy application necessitates stringent measures to safeguard data from unauthorised access and uphold patients’ rights to maintain control over their private health information and communications (45, 46). Finally, the importance of technical user-friendliness cannot be overstated, as it plays a crucial role in ensuring seamless and effective utilisation of digital health platforms (47), ultimately enhancing the overall user experience and maximising the potential benefits for patients. While our study lacked systematic data on technical support interactions and user-friendliness, it’s crucial to consider these aspects in the broader context of our study’s positive outcomes, including high levels of engagement, satisfaction, acceptability, and usability.

## 5 Conclusion

In summary, this pilot study demonstrates the feasibility and acceptability of the PAHcare™ digital platform as a promising mHealth intervention for patients with PAH. Although the clinical outcomes did not exhibit significant improvements, it is important to highlight the remarkable levels of engagement, satisfaction, acceptability, and usability experienced by participants. The platform’s noteworthy user retention and sustained engagement underscore its potential to empower PAH patients. Through accessible educational resources, personalised health insights, and direct communication channels with healthcare professionals, the platform may facilitate active patient participation in their care, foster a sense of control over their health, and contribute to an overall enhancement in well-being.

The findings of this study provide a valuable basis for future research and development of mHealth interventions targeting PAH management. Further investigation with a larger sample size, longer follow-up period, and inclusion of patients across a broad functional spectrum is warranted to evaluate the clinical benefits offered by the PAHcare™ platform comprehensively. Qualitative research can also provide a more in-depth understanding of how the newly developed PAHCare™ solution is likely to address an unmet need.

## Data availability statement

The raw data supporting the conclusions of this article will be made available by the authors, without undue reservation.

## Ethics statement

The studies involving humans were approved by the competing Ethics Committee of Hospital 12 de Octubre, Madrid, Spain, approved the study protocol V4.0 on March 22, 2022 (FPAH-CI-2101). The studies were conducted in accordance with the local legislation and institutional requirements. The participants provided their written informed consent to participate in this study.

## Author contributions

GP: Investigation, Writing – review & editing. NO: Investigation, Writing – review & editing. JD: Investigation, Writing – review & editing. AM: Investigation, Writing – review & editing. ML: Investigation, Writing – review & editing. SC: Investigation, Writing – review & editing. FL: Investigation, Writing – review & editing. SG: Investigation, Writing – review & editing. CG-G: Investigation, Writing – review & editing. PR: Investigation, Writing – review & editing. JM: Investigation, Writing – review & editing. RA: Conceptualization, Investigation, Methodology, Supervision, Writing – review & editing. HM: Data curation, Methodology, Writing – review & editing. GB: Methodology, Writing – review & editing. PE: Conceptualization, Investigation, Methodology, Supervision, Writing – review & editing.
