# Supplementary material for: Assessing the clinical benefit, safety, and patient-reported outcomes with the use of the PAHcare™ digital platform in pulmonary arterial hypertension: a pilot study
Source: Front Public Health. 2024 Feb 16;12:1335072. doi: 10.3389/fpubh.2024.1335072 (PMC10904626; doi:10.3389/fpubh.2024.1335072)

Supplementary Material

Assessing the Clinical Benefit, Safety, and Patient-reported Outcomes with the Use of the PAHcare™ Digital Platform in Pulmonary Arterial Hypertension: A Pilot Study

Gregorio Pérez Peñate, Nuria Ochoa Parra, Juan Antonio Domingo Morera, Amaya Martínez Meñaca, Marta López Ramón, Sergio Cadenas Menéndez, Fernando León Marrero, Sara Gómara de la Cal, Cristina Ghadban Garrido, Patricia Royo Tolosana, Javier Martin Puentes, Rebeca Aldonza Aguayo, Hadis Mahdavi, Gabriela Bacchini Jeanneret, Pilar Escribano Subías*[*]

***Correspondence:** Pilar Escribano Subías: pilar.escribano.subias@gmail.com

Supplementary Table 1. List of participating clinical investigation sites across Spain.

Center number	Center name and location
**01**	Hospital Universitario 12 de Octubre. Madrid, Spain
**02**	Hospital Universitario Marqués de Valdecilla. Santander, Spain
**03**	Hospital Clínico Universitario de Salamanca. Salamanca, Spain
**04**	Hospital Universitario Miguel Servet. Zaragoza, Spain
**05**	Hospital Universitario de Gran Canaria Dr. Negrín. Las Palmas de Gran Canaria, Spain

Supplementary Table 2. Health professional visits and tests performed in the CEP population.

		Baseline	Final visit	Change (Final–Baseline)
**Family doctor**				
**Number of visits**	N	5	8	4
	Mean (SD)	2.6 (1.9)	2.6 (1.8)	-1.8 (2.2)
	Median (Q1, Q3)	2.0 (2.0, 2.0)	2.5 (1.0, 3.5)	-1.0 (-3.0, -0.5)
**Tests or procedures**	N	4	8	3
	Mean (SD)	5.0 (4.7)	3.0 (2.3)	-2.0 (5.2)
	Median (Q1, Q3)	4.5 (1.0, 9.0)	2.0 (1.5, 4.0)	1.0 (-8.0, 1.0)
**Number of tests**	N	2	5	1
	Mean (SD)	9.5 (10.6)	6.8 (6.9)	2.0 (0.0)
	Median (Q1, Q3)	9.5 (2.0, 17.0)	4.0 (4.0, 5.0)	2.0 (2.0, 2.0)
**Nurse**				
**Visits**	N	11	8	7
	Mean (SD)	6.1 (7.6)	9.6 (12.0)	-2.9 (6.4)
	Median (Q1, Q3)	4.5 (1.0, 6.5)	6.0 (4.0, 10.0)	-1.0 (-3.0, 0.0)
**Tests or procedures**	N	6	6	2
	Mean (SD)	2.7 (1.0)	3.7 (1.2)	0.5 (0.7)
	Median (Q1, Q3)	3.0 (2.0, 3.0)	3.0 (3.0, 4.0)	0.5 (0.0, 1.0)
**Number of tests**	N	4	3	1
	Mean (SD)	15.3 (14.2)	11.3 (4.7)	0.0 (0.0)
	Median (Q1, Q3)	12.5 (6.5, 24.0)	13.0 (6.0, 15.0)	0.0 (0.0, 0.0)
**Pulmonologist**				
**Number of visits**	N	16	10	12
	Mean (SD)	4.3 (5.5)	5.0 (6.9)	-0.8 (9.2)
	Median (Q1, Q3)	2.0 (2.0, 5.0)	2.0 (2.0, 5.0)	0.0 (-2.5, 1.0)
**Public or private**	Public	18	10	-
	Private	0	1	-
**Tests or procedures**	N	14	8	7
	Mean (SD)	3.3 (2.1)	3.5 (2.2)	-0.6 (1.1)
	Median (Q1, Q3)	3.5 (1.0, 5.0)	2.5 (2.0, 5.5)	0.0 (-2.0, 0.0)
**Number of tests**	N	4	5	1
	Mean (SD)	13.8 (17.2)	6.8 (5.4)	2.0 (0.0)
	Median (Q1, Q3)	7.5 (3.5, 24.0)	5.0 (2.0, 11.0)	2.0 (2.0, 2.0)
**Cardiologist**				
**Number of visits**	N	12	11	6
	Mean (SD)	2.5 (1.8)	2.3 (1.6)	-0.3 (2.6)
	Median (Q1, Q3)	2.0 (1.0, 3.0)	2.0 (1.0, 3.0)	0.0 (-1.0, 2.0)
**Tests or procedures**	N	9	8	4
	Mean (SD)	3.7 (2.1)	3.9 (2.0)	-0.3 (3.1)
	Median (Q1, Q3)	3.0 (2.0, 6.0)	3.5 (2.5, 5.5)	-1.0 (-2.5, 2.0)
**Number of tests**	N	3	4	2
	Mean (SD)	5.3 (4.0)	5.8 (4.4)	-0.5 (9.2)
	Median (Q1, Q3)	3.0 (3.0, 10.0)	6.0 (2.0, 9.5)	-0.5 (-7.0, 6.0)

CEP, cost-evaluable population; Q1, Q3, first and third quartile; SD, standard deviation.

Supplementary Table 3. Outpatient visits, emergency, and other specialists' visits in the CEP population.

		Baseline	Final visit	Change (Final–Baseline)
**Hospital outpatient visits**				
**Number of visits**	N	5	1	2
	Mean (SD)	3.6 (2.9)	6.0 (0.0)	-3.5 (4.9)
	Median (Q1, Q3)	3.0 (2.0, 6.0)	6.0 (6.0, 6.0)	-3.5 (-7.0, 0.0)
**Tests or procedures**	N	1	1	0
	Mean (SD)	5.0 (0.0)	6.0 (0.0)	-
	Median (Q1, Q3)	5.0 (5.0, 5.0)	6.0 (6.0, 6.0)	-
**Number of tests**	N	1	1	0
	Mean (SD)	8.0 (0.0)	8.0 (0.0)	-
	Median (Q1, Q3)	8.0 (8.0, 8.0)	8.0 (8.0, 8.0)	
**Emergency room visits**				
**Visits**	N	2	3	0
	Mean (SD)	1.5 (0.7)	1.0 (1.0)	-
	Median (Q1, Q3)	1.5 (1.0, 2.0)	1.0 (0.0, 2.0)	-
**Tests or procedures**	N	1	2	0
	Mean (SD)	1.0 (0.0)	2.5 (0.7)	-
	Median (Q1, Q3)	1.0 (1.0, 1.0)	2.5 (2.0, 3.0)	-
**Number of tests**	N	1	2	0
	Mean (SD)	2.0 (0.0)	2.5 (0.7)	-
	Median (Q1, Q3)	2.0 (2.0, 2.0)	2.5 (2.0, 3.0)	-
**Another specialist**				
**Number of visits**	N	5	7	3
	Mean (SD)	2.2 (0.4)	1.0 (0.6)	-0.7 (0.6)
	Median (Q1, Q3)	2.0 (2.0, 2.0)	1.0 (1.0, 1.0)	-1.0 (-1.0, 0.0)
**Public or private**	Public	0	7	-
	Private	0	1	-
**Tests or procedures**	N	3	4	2
	Mean (SD)	3.3 (1.5)	2.0 (0.8)	-1.5 (0.7)
	Median (Q1, Q3)	3.0 (2.0, 5.0)	2.0 (1.5, 2.5)	-1.5 (-2.0, -1.0)

CEP, cost-evaluable population; Q1, Q3, first and third quartile; SD, standard deviation.

Supplementary Figure 1. Proportion of patients engaging with disease-specific knowledge and management magazines. Blue areas reflect the proportion who read the content and grey areas the proportion who did not access the corresponding content.

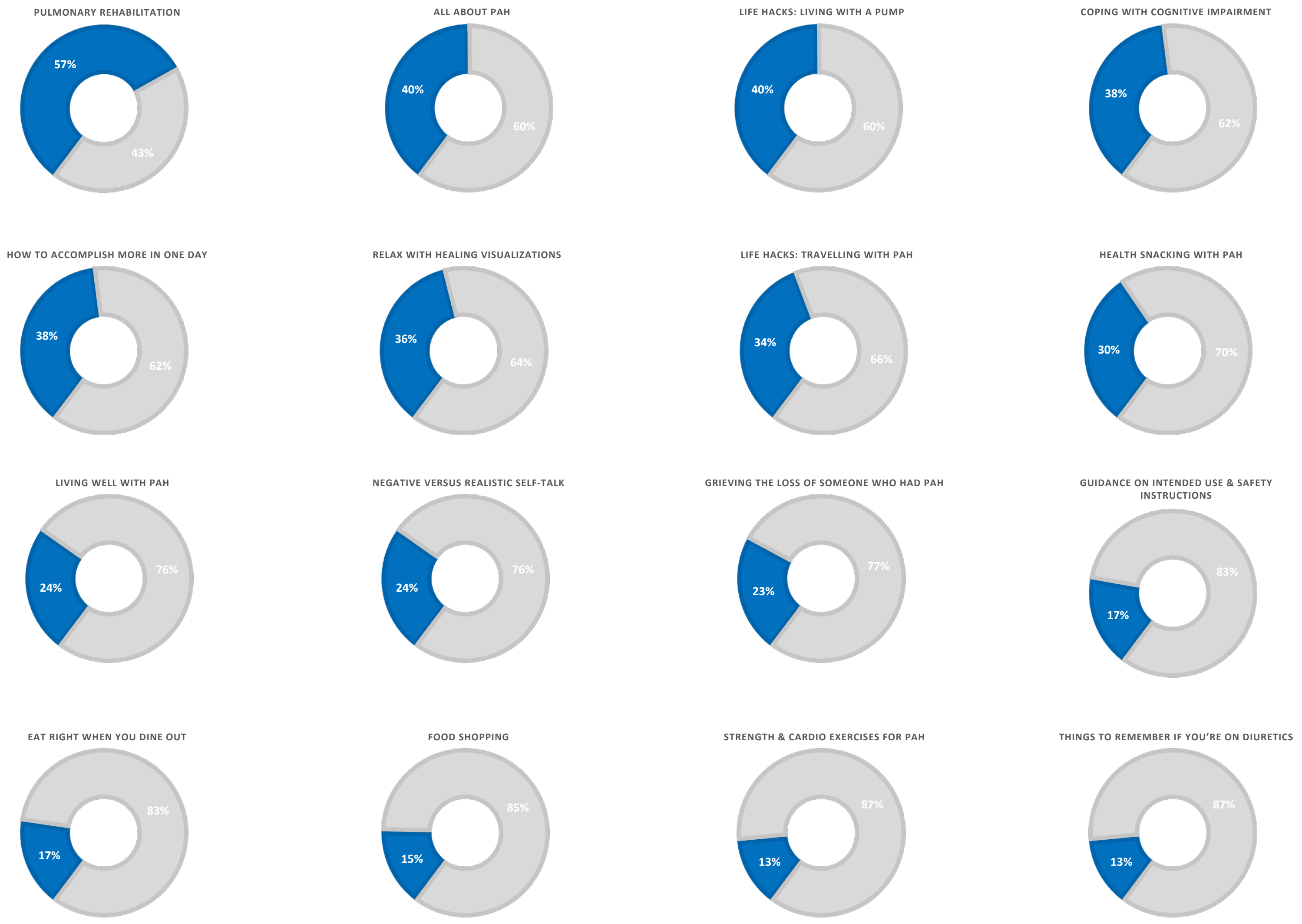

Supplementary Figure 2 (continued). Proportion of patients engaging with disease-specific knowledge and management magazines. Blue areas reflect the proportion who read the content and grey areas the proportion who did not access the corresponding content.

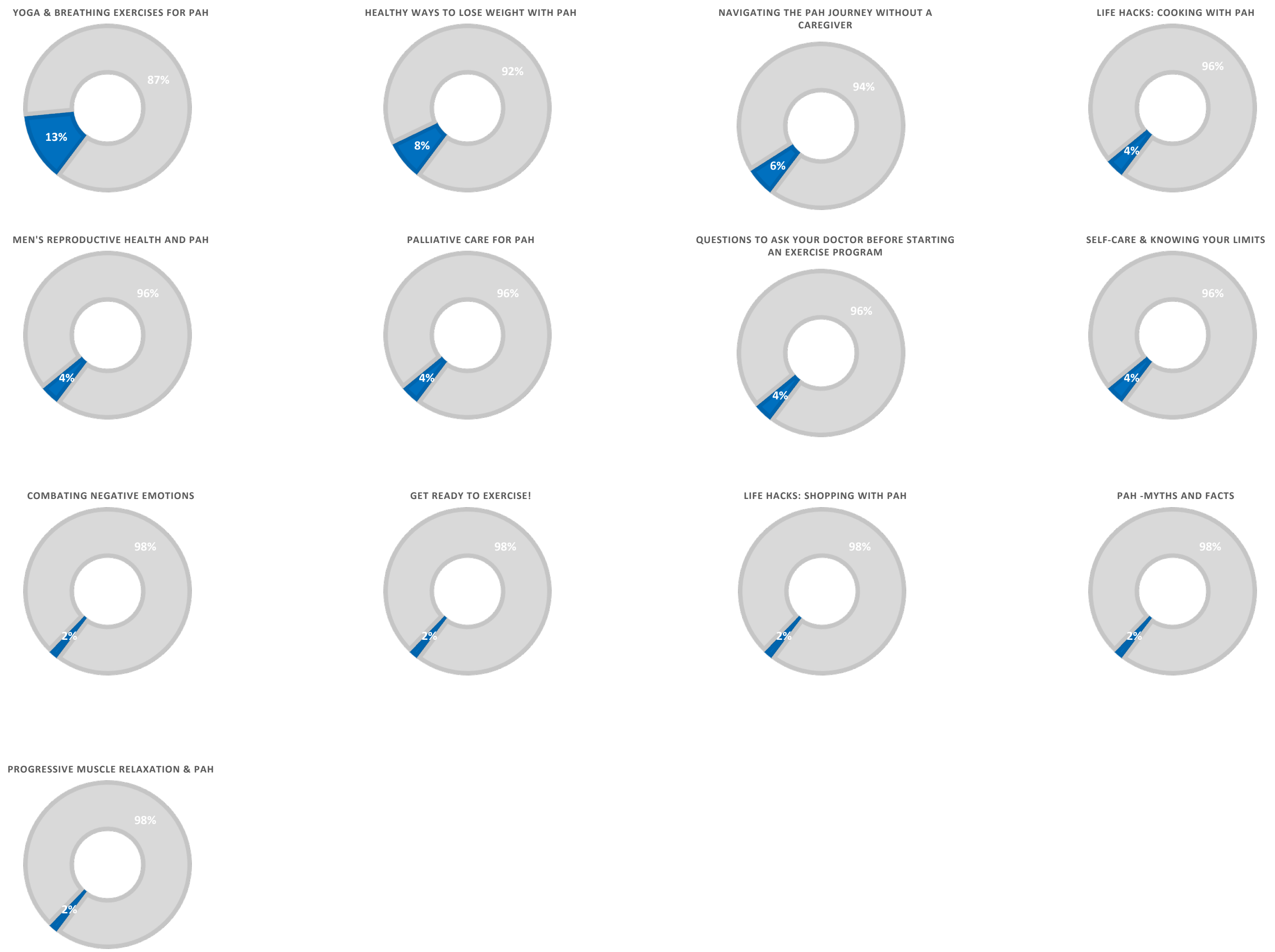

Supplementary Figure 2. Proportion of patients accessing the platform's structured educational pathway and progression to subsequent levels. Red areas reflect the proportion who read the content and grey areas the proportion who did not access the corresponding content.

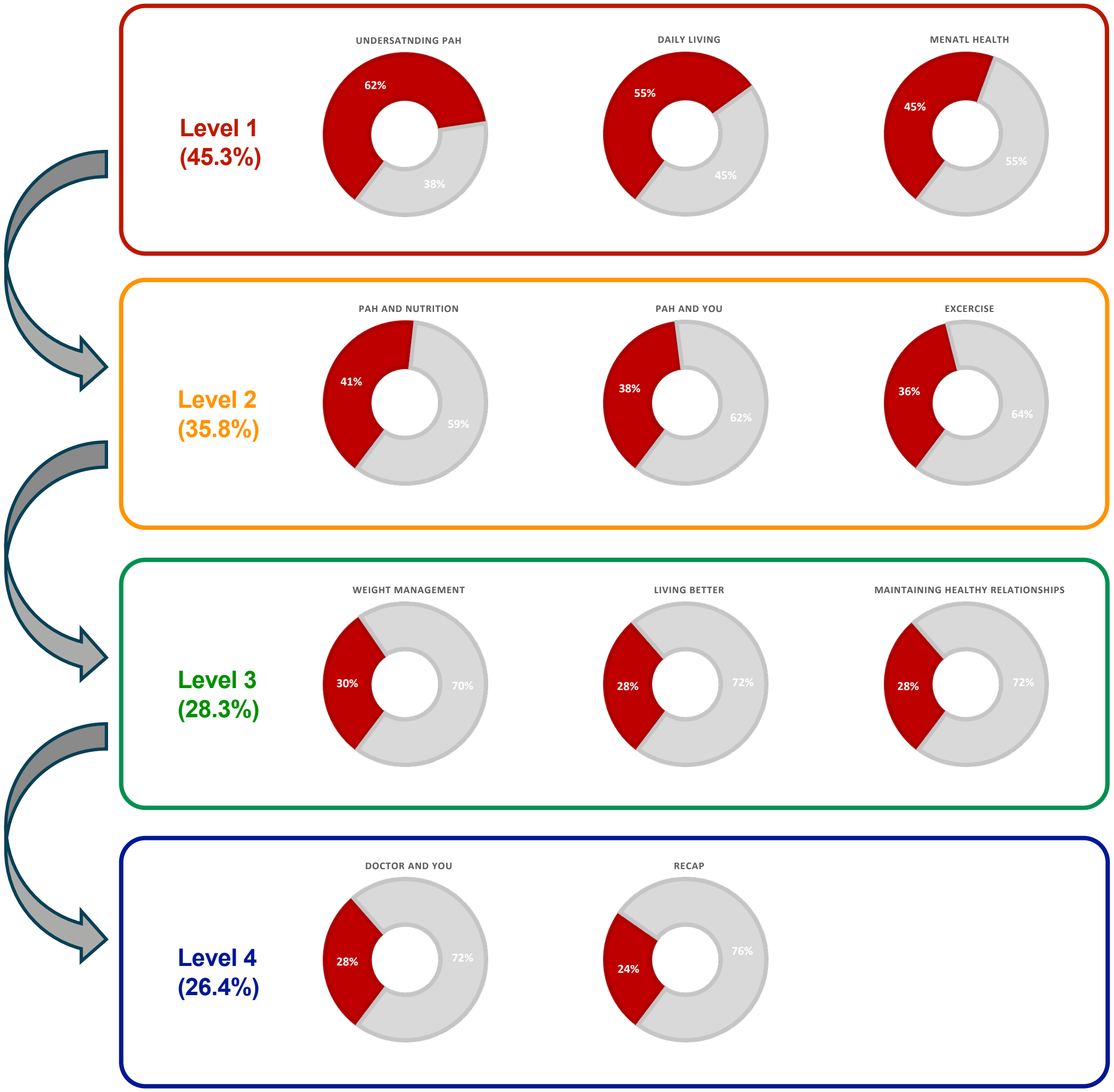

Supplement: Supplementary file 1 [file Data_Sheet_1.PDF]
